# AI-Y: An AI Checklist for Population Ethics Across the Global Context

**DOI:** 10.1007/s40471-025-00362-w

**Published:** 2025-07-09

**Authors:** Yulin Hswen, John A. Naslund, Margaret Hurley, Bart Ragon, Margaret A. Handley, Fang Fang, Emily E. Haroz, Joyce Nakatumba-Nabende, Alastair van Heerden, Elaine O. Nsoesie

**Affiliations:** 1 Department of Epidemiology and Biostatistics and Medicine, University of California, San Francisco, USA; 2 Department of Global Health and Social Medicine, Harvard Medical School, Boston, USA; 3 Health Sciences Library, University of Virginia, Charlottesville, USA; 4 Karolinska Institute, Stockholm, SE Sweden; 5 Center for Indigenous Health, Department of International Health; Center for Suicide Prevention, Department of Mental Health, Johns Hopkins Bloomberg School of Public Health, Baltimore, USA; 6 Department of Computer Science, Makerere University, Kampala, UG Uganda; 7 Department of Paediatrics, University of the Witwatersrand, Johannesburg, ZA South Africa; 8 Department of Global Health, Boston University School of Public Health, Boston, USA

**Keywords:** Artificial Intelligence, Population Health, Ethics, Accountability, Transparency, Public Health, Digital Health, AI Governance

## Abstract

**Purpose of Review:**

The goal of this narrative review is to introduce and apply *Hswen’s AI Checklist (AI-Y) for Population Ethics*, a structured ethical framework created to evaluate the development and deployment of artificial intelligence (AI) technologies in public health. The review addresses key questions: How can AI be ethically assessed across global healthcare contexts and what principles are needed to ensure contextually appropriate AI use in population health.

**Recent Findings:**

Recent research highlights a significant disconnect between AI development and ethical implementation, especially in low-resource settings. Studies reveal issues such as homogeneity in the training data, and limited accessibility. Through six global case studies—spanning dementia care in Sweden, environmental forecasting in Europe, suicide prevention in Native American communities, schizophrenia care in India and the U.S., and cervical cancer and tuberculosis diagnosis in Low- and Middle-Income Countries—researchers demonstrate AI’s promise in enhancing preparedness diagnosis, screening, and care delivery while also underscoring ethical gaps in accountability, and governance.

**Summary:**

Our examination using the AI-Y Checklist found that ethical blind spots are widespread in the development and deployment of AI tools for population health—particularly in areas of model generalizability, accountability, and transparency of AI decision-making. Although AI demonstrates strong potential to enhance disease detection, resource allocation, and preventive care across diverse global settings, most systems evaluated in our six case studies did not meet key ethical criteria such as access, and localized validation and development. The major takeaway is that technical excellence alone is insufficient; ethical alignment is critical to the responsible implementation of AI in public health. The AI-Y Checklist provides a scalable framework to identify risks, guide ethical decision-making, and foster global accountability. For future research, this framework enables standardized evaluation of AI systems, encourages community co-design practices, and supports the creation of policy and governance structures that ensure AI technologies advance health ethics.

## Introduction

The rapid advancement of Artificial Intelligence (AI) and Machine Learning (ML) technologies has raised concerns about the transparency, reproducibility, and scientific rigor of these systems. Efforts are underway to establish standards, frameworks, and guidelines to ensure the development of accountable and scientifically sound AI and ML tools [1, 2]. Despite these efforts, significant gaps remain between industry practices and academic research, underscoring challenges for this rapidly evolving field [3].

For individuals not directly involved in the technical development of AI or ML tools, evaluating potential risks and limitations can be difficult due to inconsistent or insufficient information. The level of detail provided about a tool often varies, with scholarly articles focusing on efficacy in specific healthcare applications while overlooking critical information about development processes, the underlying data, training methodologies, or validation tests. Proprietary software, constrained by intellectual property and commercial considerations, often lacks transparency regarding its data models or algorithms. Even open-source tools may vary significantly in their documentation, leaving gaps in understanding how these systems are developed and applied.

AI has significant potential to advance population health by improving disease diagnosis, optimizing treatment protocols, enhancing disease surveillance, accelerating drug discovery, and streamlining resource allocation. To fully realize the potential of AI in improving population health, it is essential to focus on solutions that address the unique needs of different communities. This requires developing tools based on locally relevant data, testing them in the environments where they will be used, and ensuring their accessibility and affordability [4]. Regular evaluations and audits are also necessary to identify and minimize unintended harms while maximizing the benefits for diverse populations. By integrating these principles into the design and implementation of AI technologies, public health systems can harness these innovations to drive measurable improvements in health outcomes across populations.

Recent research highlights a disconnect between AI development and real-world implementation, leading to potential unintended consequences or misapplications of these tools [5]. We propose adopting structured AI ethical evaluation criteria developed here by Hswen and colleagues that can help guide the responsible use of studying and deploying AI in applied settings. Hswen’s AI Checklist (AI-Y) for Population Ethics aims to ensure rigorous testing, appropriate validation, and alignment with population health objectives during the development and/or implementation of AI systems [6].

### AI Checklist (AI-Y) for Population Ethics

**Fig. 1:**
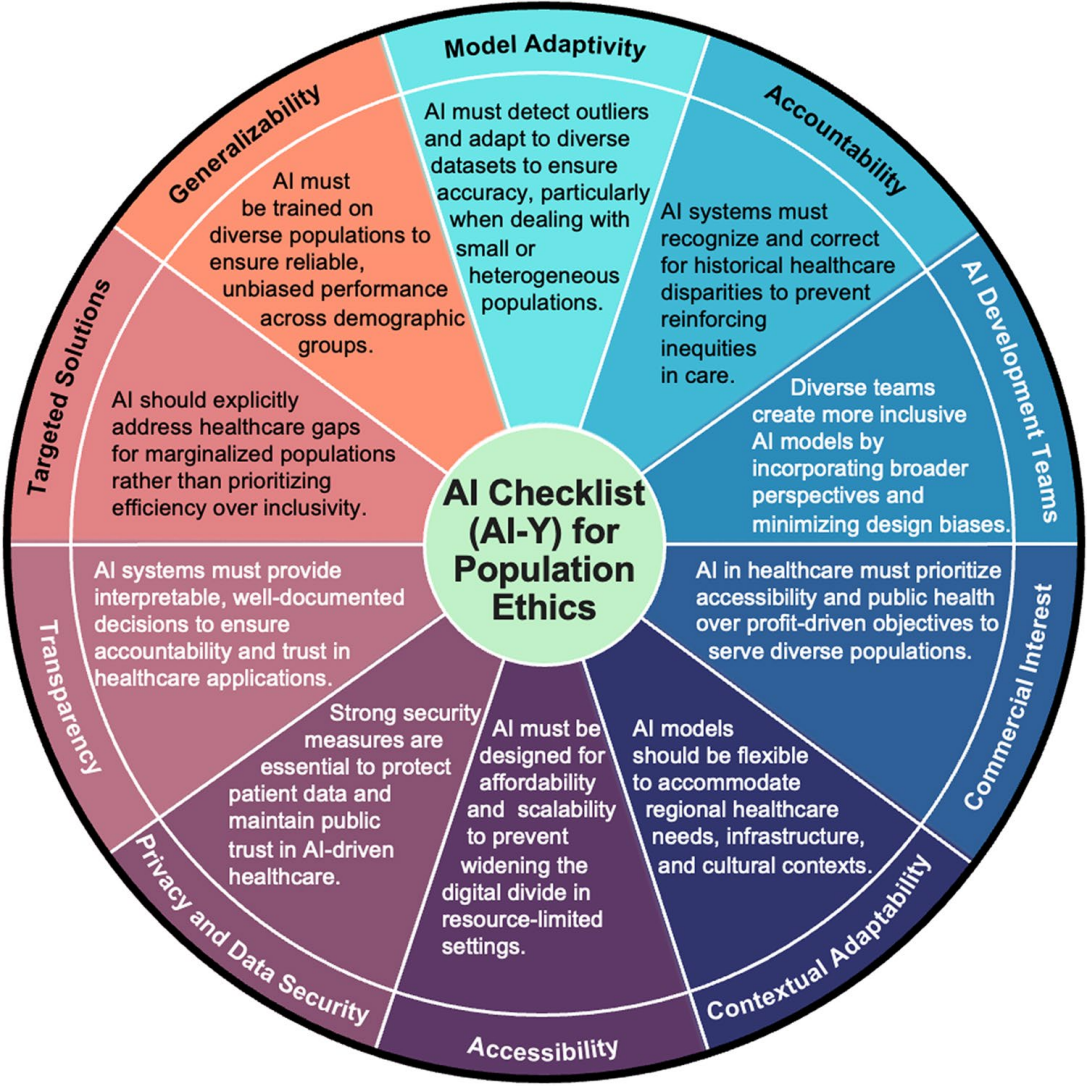
Hswen's AI Checklist (AI-Y) for Population Ethics

As AI technologies become increasingly integrated into healthcare and public health systems, ensuring their effectiveness, reliability, and ethical deployment at a population-wide scale is critical. The proposed criteria shown in Table 1. provide a structured framework for evaluating AI systems used in health applications, ensuring that they function optimally across populations while minimizing potential harms. The need for this assessment framework is based on the following key considerations (seen in Fig. 1.):

1. Model Adaptivity.
  AI must detect outliers and adapt to diverse datasets to ensure accuracy, particularly when dealing with small or heterogeneous populations.
2. Accountability.
  AI systems must recognize and correct for historical healthcare disparities to prevent reinforcing inequities in care.
3. AI Development Teams.
  Diverse teams create more inclusive AI models by incorporating broader perspectives and minimizing design biases.
4. Commercial Interest Assessment.
  AI in healthcare must prioritize accessibility and public health over profit-driven objectives to serve diverse populations.
5. Contextual Adaptability.
  AI models should be flexible to accommodate regional healthcare needs, infrastructure, and cultural contexts.
6. Accessibility.
  AI must be designed for affordability and scalability to prevent widening the digital divide in resource-limited settings.
7. Privacy and Data Security.
  Strong security measures are essential to protect patient data and maintain public trust in AI-driven healthcare.
8. Transparency.
  AI systems must provide interpretable, well-documented decisions to ensure accountability and trust in healthcare applications.
9. Targeted Solutions.
  AI should explicitly address healthcare gaps for marginalized populations rather than prioritizing efficiency over inclusivity.
10. Generalizability.
  AI must be trained on diverse populations to ensure reliable, unbiased performance across demographic groups.

**Table 1:** AI case studies assessed using the AI-Y Checklist

AI Study		Case 1: AI in Dementia Care		Case 2: AI and Machine Learning for Environmental Challenges: Implications for Public Health		Case 3: Artificial Intelligence in Suicide Risk Identification		Case 4: AI for Cervical Cancer Screening		Case 5: Digital Tools and AI/ML for Relapse Prevention in Schizophrenia		Case 6: AI in Tuberculosis Diagnosis	
Author		Fang Fang, MD, PhD		Margaret Handley, PhD, MPH		Emily E. Haroz, PhD, MHS, MA		Joyce Nakatumba-Nabende, PhD, MSc		John Naslund, PhD		Alastair Van Heerden, PhD	
Location		Sweden		Europe		United States		LMICs		United States and India		LMICs	
Topic	Criteria												
1. Model Adaptivity	AI must detect outliers and adapt to diverse datasets to ensure accuracy, particularly when dealing with small or heterogeneous populations	U	This case used data from the Swedish National Study on Aging and Care (SNAC) study. According to the exclusion criteria listed, the study sample is not likely representative of the general Swedish population at risk for dementia	U	The training data is from the ENS of the European Centre for Medium-Range Weather Forecasting (ECMWF). It is a reanalysis of data for 40 years covering 1979–2018. There is no discussion of how data varies from region to region or for different environmental factors (e.g. urban vs rural, areas with limited satellite images or other forms of data)	S	The final model that is being rolled out is a model that has been re-trained on data specific to the racial population and context that it will be implemented in	U	The training data is collected from a Ugandan population of women across the age groups 25–49	U	The use of AI/ML was exploratory. This is important area for future exploration	U	Teams aim to avoid bias adapt to diverse datasets by doing validation studies in both high income and low income settings across various population
2. Accountability	AI systems must recognize and correct for historical healthcare disparities to prevent reinforcing inequities in care	NS	This case does not address historical accountability. However, given the universal healthcare system in Sweden, healthcare disparities are not likely a major concern	U	The training data is not discussed in detail. The paper does not discuss known concerns with these types of data, such as variability in cloud cover in different regions, lack of data collection (e.g. sensor data) altogether for some regions, changes in measurement over time, etc	S	Yes, we used data specific to a population that has been left out of other tool development work. The modeling work was developed over years, and builds upon a 40 + year trust-partnership between an academic university and a Tribal nation	S	The dataset aims to cover a Ugandan population that is unrepresented with the available open source datasets that have been reported in available research papers	NS	The data likely does not account for this	S	The AI addresses historical accountability
3. AI Development Teams	Diverse teams create more inclusive AI models by incorporating broader perspectives and minimizing design biases	NS	There is little information in this regard. The development team is not greatly representative of diverse backgrounds	NS	The development team are all from Google DeepMind, London, UK	S	The development team includes representatives from diverse disciplines, and from the population the model is intended to be used with	S	The team is diverse and consists of ML researchers from Makerere University, as well as health specialists, gynecologists, and nurses from the Uganda Cancer Institute	S	The development team was diverse, with contributors from both the U.S. and India. Patients, family members, and clinicians were also engaged throughout the research project	NS	The majority of technical expertise and algorithm development originates in high-income countries. Validation and evaluation studies include LMIC colleagues, but the extent to which they are equal intellectual partners varies
4. Commercial Interest Assessment	AI in healthcare must prioritize accessibility and public health over profit-driven objectives to serve diverse populations	U	The authors declare that the research was conducted in the absence of any commercial or financial relationships that could be construed as a potential conflict of interest	U	This is a commercial product in that the algorithm is up for several patents and the authors all have stock invested in the related company. The data used is publicly available. The focus of this work is to create weather predictions that are accurate for 15 day forecasts that will help with improving preparedness. There is no mention of ethics or fairness in the stated goals	S	There are no commercial interests for this model	S	There are no commercial interests	S	There are no commercial interests	U	Some initiatives prioritize ethics, yet commercial motivations can overshadow fairness goals
5. Contextual Adaptability	AI models should be flexible to accommodate regional healthcare needs, infrastructure, and cultural contexts	S	The AI can likely be adapted in Sweden and in other high-income settings	U	It is not clear if the models can be adjusted for different contexts and populations	S	The model was re-trained in the specific cultural context, and its implementation is being guided by community voices and local providers	S	The model has been re-trained to cover datasets from four different cultural contexts in Uganda	S	The AI was used across diverse settings, demonstrating potential for cross cultural application	NS	The goal is for the AI to be adaptable to contextual settings, but this is currently limited. For example, during the CAD4 TB study, fuel restrictions during unrest prevented generators from powering the X-ray machine
6. Accessibility	AI must be designed for affordability and scalability to prevent widening the digital divide in resource-limited settings	U	Potentially yes and more so than other existing prediction models, as it does not require neuroimaging. However, electronic health care records, ECG, self-reported information and mental rotation tests are included in the final algorithm	NS	There is no mention of these considerations, but the data sources are publicly available	U	The model is accessible to a low-resource setting in the U.S. with hopes it could generalize to similar settings both domestically and globally	S	The technology was used in LMIC settings and can be scaled to these contexts. There is accessibility and uptake in cancer screening clinics in low-resource settings	S	The technology was used in LMIC settings. However, there are still challenges with accessibility and uptake in low-resource settings	S	The AI is accessible in LMICs
7.Privacy and Data Security	Strong security measures are essential to protect patient data and maintain public trust in AI-driven healthcare	S	As this case used data from the SNAC, there are strong measures in place to safeguard sensitive patient data	U	This work does not apply patient data, so this is not applicable	S	The model will be entirely implemented on local servers that sit behind the health facilities firewalls. Data only leaves this system for the purposes of a clinical trial and under a research protocol	S	The research followed strict data protection protocols during the model development and evaluation. The image data is anonymized before model development. However, it has not been fully evaluated for use in routine care settings	S	The research followed strict data safety protocols. However, use in routine care settings has not yet been achieved, which would require similar strict data safety measures	U	Regulatory standards exist, but the degree of robust, universally applied data protection varies
8.Transparency	AI systems must provide interpretable, well-documented decisions to ensure accountability and trust in healthcare applications	S	The AI model is transparent	NS	The model's processes and outputs are not currently defined in the case study	S	The output of the model has been guided by local providers and community members. The electronic health informatics infrastructure is limited, but a clinical decision support website is being developed to promote transparency	S	The data and algorithms are available open source	S	All data and algorithms are available open source	S	X-rays are labeled based on the algorithm's grading of potential pulmonary TB
9. Targeted Solutions	AI should explicitly address healthcare gaps for marginalized populations rather than prioritizing efficiency over inclusivity	NS	The AI model has not been tested in marginalized populations. Given the characteristics of the SNAC study, whether it serves marginalized populations remains to be tested	NS	The AI model does not serve vulnerable populations	S	The model is specifically designed for American Indian/Alaska Native populations—racial groups that face the largest suicide-related inequities	S	The focus is on women in LMICs across different and diverse backgrounds, some of whom are also from rural populations	S	The focus is on persons living with schizophrenia, which represents a highly vulnerable/marginalized patient population	S	Community- and facility-based screening for active cases, which eliminates the need for patients to leave and return, addresses a significant need
10. Generalizability	AI must be trained on variations populations to ensure reliable, unbiased performance across demographic groups	NS	A heterogenous sample population was not used	U	This is not clear, since the data sources are from an existing data repository which does not address the heterogeneity of the data, data training biases, or changes over time in the data quality and comprehensiveness	S	Because we re-trained a model that was developed externally, the full model was trained and validated on multiple heterogenous samples	S	The model was trained and validated based on mobile colposcopy images from different and diverse groups in Uganda	S	The study involved a heterogeneous sample, drawing from one site in the U.S. and two sites in India. There may be biases in the data, as the use of AI/ML was exploratory	U	This work is ongoing

S – Satisfactory

NS- Not Satisfactory

U—Unknown

A narrative review was selected to synthesize a diverse and complex body of evidence on a topic that remains relatively underexplored [7]. Unlike systematic reviews, which are optimal for narrowly defined questions and methodologically uniform studies [8], the narrative approach accommodates heterogeneity across study designs, contexts, and outcomes. This methodology is particularly well-suited to the present analysis, which spans multiple geographic regions, includes a wide range of AI systems, and examines varied health domains. The six case studies included in this narrative review were identified based on geographic diversity—North America, Europe, Asia, and Africa, representation of multiple disease categories—including infectious, non-communicable, and behavioral health conditions and heterogeneity in AI system types—machine learning for predictive analytics, natural language processing for clinical applications, computer vision for diagnostics, and deep learning for environmental modeling, ensuring a comprehensive and globally representative evaluation of AI applications in public health.

The primary objective of this review is to critically evaluate how AI is being deployed to enhance population health, using an ethical framework applied across six international case studies. Each study is assessed through key ethical dimensions—data integrity, transparency, adaptability, and accessibility—to examine the responsible use of AI in healthcare and environmental settings. The review highlights how AI implementation is shaped by contextual factors such as data representativeness, regulatory environments, and commercial incentives. Authors reviewed each study using the AI-Y Checklist to assess whether it met the specified criteria. Each item was marked as *Satisfactory (S) or Not Satisfactory (NS).* If a criterion was not explicitly addressed—for example, no mention of commercial interest—it was marked as *Unknown (U).* Through these examples, the review underscores AI’s potential to improve healthcare delivery, research precision, and system efficiency, while also addressing critical challenges such as algorithmic reliability, scalability, and data security. This synthesis provides a comprehensive and ethically grounded understanding of AI's evolving role in advancing global public health.

## AI in Dementia Care

### Background

Dementia is a major public health challenge due to the large numbers of people affected and the current lack of a clear path to prevention or cure [9]. AI is increasingly being integrated into dementia care worldwide, advancing disease detection, enabling personalized treatment, and improving healthcare efficiency [10]. However, ethical challenges such as privacy, bias, and transparency must be addressed to maximize AI's potential while minimizing risks.

### Challenges in Early Diagnosis of Dementia

Early diagnosis of dementia remains challenging due to its long prodromal phase, which includes both cognitive deficits and various noncognitive symptoms [11, 12]. Traditional Diagnostic Methods include cognitive assessments, neuroimaging, and biomarker analysis are valuable but costly, invasive, and dependent on specialized personnel [13–15]. These methods often fail to account for individual variations in genetics, environment, and lifestyle, limiting their applicability across diverse populations [16].

### Accessibility Barriers

Regions with healthcare variances experience greater obstacles in accessing diagnostic technologies [17]. High costs and limited scalability hinder widespread implementation, reducing the effectiveness of these methods for population screening [18]. In addition, delayed detection limits early intervention, exacerbating the disease’s impact on patients, families, and society.

### AI’s Role in Advancing Dementia Diagnosis and Treatment

AI offers promising solutions to overcome existing diagnostic and treatment challenges. For example, it offers enhanced diagnostic accuracy. ML and natural language processing improve the accuracy of dementia diagnosis by analyzing complex, multimodal datasets from diagnostic tests, electronic health records, and mobile devices [19–21]. AI-driven remote diagnostics also improves accessibility, reducing reliance on specialized care, enabling mass screening and decreasing health variations in dementia care [22]. AI can also personalize treatment and tailor interventions to individual patient needs, facilitating precision medicine and personalized care approaches [23]. These advancements optimize resource use, allowing healthcare providers to focus on patient-centered care.

### Example of AI Integration in Europe: The Prominent Project

AI-powered solutions in dementia care are gaining traction in Europe. The Prominent Project (Karolinska Institute, Sweden) is a collaborative initiative between academia, healthcare, the pharmaceutical industry, and medical device companies across Europe. It aims to create a digital decision support system for precision medicine in dementia care.

### Ethical and Methodological Challenges

Despite AI’s benefits, several challenges must be addressed for responsible integration. Data privacy and security concerns arise due to the sensitive nature of personal data necessitates robust legal protections to prevent unauthorized use and breaches. Algorithm validity and bias present another challenge, as AI models are susceptible to errors and biases common in observational studies, requiring rigorous validation. Additionally, transparency and accountability must be considered, as many AI systems operate as opaque"black boxes,"complicating decision validation and hindering generalization to diverse settings.

## AI and ML for Environmental Challenges

### Background

AI-based solutions are increasingly applied to environmental challenges, including weather forecasting, resource management, natural hazards, and climate change, with significant implications for public health. Recent reports highlight AI’s role in predicting climate risks, enhancing preparedness, and protecting lives, property, and livelihoods on a global scale [24]. However, as AI models evolve, questions arise about how fairness principles are incorporated into guidelines and how researchers and the public can trust ML-generated outcomes [25]. From a global health perspective [26], it is essential to ensure AI fairness, appropriateness, and reliability in climate and environmental applications.

### Current Methods for Weather and Climate Prediction

Traditional numerical weather prediction (NWP) relies on physics-based equations to simulate atmospheric conditions and generate forecasts. While effective, these models face limitations, such as limited use of historical data to refine model accuracy, and challenges in assimilating real-time atmospheric changes, leading to delayed or less precise predictions. There are also data quality concerns, including imprecision in key equations and difficulties in capturing rapidly evolving extreme weather conditions.

Despite these constraints, traditional models remain foundational to global weather prediction, but their limitations highlight the need for AI-driven innovations.

### Gaps That Need to Be Filled

Several gaps must be addressed to improve AI and ML for environmental challenges. Traditional weather models struggle with error-prone forecasting, often failing to predict extreme events with high resolution and real-time adaptability. Additionally, many models exclude critical data from underrepresented geographic areas, such as rural, remote, and low-resource regions. Furthermore, a lack of cross-disciplinary integration poses a challenge, as environmental AI research often lacks input from local experts and public health professionals, limiting its real-world effectiveness.

### How AI is Filling Those Gaps

AI-powered weather and climate models are transforming environmental forecasting, offering enhanced predictive accuracy and broader data integration [27, 28]. ML-based weather forecasting enables the reanalysis of historical and current atmospheric conditions, predicting hundreds of weather variables worldwide with high-resolution accuracy. AI has also improved early warning systems for extreme events, demonstrating greater accuracy in forecasting tropical cyclones, atmospheric rivers, and extreme temperature events. Additionally, automated environmental monitoring through AI-driven satellite and radar analysis enhances ecosystem tracking, ensuring environmental justice through deforestation and wildfire risk assessments [29]. These models offer scalability and adaptability, and can rapidly adjust predictions as new data becomes available, outperforming static traditional models. AI plays a crucial role in public health by enhancing climate monitoring, which assists global health preparedness, particularly in predicting vector-borne disease outbreaks linked to climate change.

### Ethical Issues of AI in Environmental Forecasting

Data gaps and exclusion bias arise when AI models rely on densely populated regions for data inputs, leading to uncertainty for rural and resource-limited areas [29]. Algorithmic trust and transparency are key. AI models must clearly communicate uncertainties, ensuring public trust in AI-driven climate predictions [29]. Additionally, AI tools must include input from affected communities to ensure local representation in model development, preventing external decision-making from dominating local realities [30]. Data ownership and access should also be considered. There is a need for fair distribution of computational resources, ensuring global access to AI-driven environmental models. Lastly, effective AI implementation requires scientific convergence and collaboration, integrating natural, social, and computational sciences to enhance model effectiveness, particularly in public health applications [24].

### Strategies to Address Ethical and Implementation Challenges

To ensure geographic and demographic representation, AI models should be trained on diverse, global datasets to reduce regional prediction gaps [31]. Open science and resource sharing is also critical, as public access to computational models can increase inclusivity in AI research and environmental forecasting. Additionally, advancing trustworthy AI in climate science is essential. AI models should be interpretable and transparent, ensuring stakeholder confidence in AI-generated climate predictions. [29]

## AI in Suicide Risk Identification

### Background

AI-supported clinical decision tools have the potential to improve suicide risk identification, particularly when developed in collaboration with communities facing significant health variations. Suicide remains a critical global public health challenge, with an estimated 726,000 deaths annually [32]. Indigenous populations experience disproportionately higher suicide rates [33], with American Indians and Alaska Natives (AI/ANs) in the U.S. having nearly double the national average suicide rate (27.1 vs. 14.2 per 100,000 in 2022) [34]. Identifying individuals at risk is essential for prevention, yet current suicide risk assessment tools have limitations and lack population-specific validation [35–37]. No models have been developed specifically for AI/AN communities, exacerbating gaps in effective prevention strategies.

### Challenges in Suicide Risk Identification

Suicide risk assessment methods include clinician evaluations, brief screening tools, and in-depth risk assessments, each with inherent limitations. Clinician assessments are considered the gold standard but require specialized training [35, 38] and their accuracy varies, with potential for subjective inconsistencies across providers. Brief screening tools are widely used but face challenges in implementation and may lack generalizability across diverse populations [39–41]. In-depth risk assessments are recommended in clinical settings but have uncertain accuracy advantages over brief tools [42]. Most conventional screening tools rely on a limited set of risk factors combined in manually administered assessments [43]. Given the complex and multifactorial nature of suicide risk, these approaches may be insufficient, particularly in rural and under-resourced communities. For AI/AN populations, frequent provider turnover in rural settings and the absence of culturally validated risk assessments further impede effective risk identification.

### AI-Driven Approaches to Suicide Risk Identification

AI models utilizing electronic health record data present a promising solution to these challenges [44–48]. AI-based methods offer higher predictive accuracy, outperforming traditional tools by analyzing large-scale, longitudinal datasets. They also offer improved implementation feasibility as they reduce dependence on manual screening processes, easing resource burdens in understaffed or rural clinics.

### Potential Risks and Ethical Considerations

High false-positive rates can overburden clinical resources and undermine trust in AI predictions [49, 50]. In addition, algorithmic distortions may arise if AI models are trained on non-representative data or fail to consider cultural and contextual nuances. Lack of transparency in AI models also raises concerns about clinical trust and interpretability in suicide prevention efforts. To ensure effective and ethical AI implementation in AI/AN communities, frameworks emphasizing participatory research, representative data, governance structures, and culturally responsive care are essential.

### Community-Guided AI: The Native-RISE Project

A successful case study demonstrating these principles is the Native-RISE (Risk Identification for Suicide and Enhanced Care) project, initiated in 2017 through a collaboration between Tribal leaders and the Johns Hopkins Center for Indigenous Health (JHCIH). Tribal partners sought AI-enhanced risk identification to expand suicide prevention efforts in geographically isolated communities [51, 52], and existing clinical teams faced significant logistical challenges in reaching high-risk individuals. JHCIH developed and implemented a predictive AI model leveraging 10 years of local community-based data [53]. The model was guided by local practitioners [54], prospectively tested to assess real-world effectiveness, and implemented within Tribal health systems to enhance risk detection. As a result, the AI-driven approach expanded outreach and improved the identification of high-risk individuals [46]. To refine the model and apply it to the health system, researchers further tested existing suicide risk models for transportability [55] and re-trained the most effective one using locally specific data [56, 57]. The re-trained model significantly outperformed existing risk assessment methods, achieving an AUC of 0.83, indicating high predictive accuracy, compared to standard practices, which had an AUC of 0.64. The model’s development and deployment were overseen by a community advisory board, local providers, and patients, ensuring cultural appropriateness and accountability [58]. This approach helped reduce predictive distortions and addressed concerns about algorithmic reliability.

## AI for Cervical Cancer Screening

### Background

Cervical cancer remains one of the most prevalent cancers in LMICs, particularly in sub-Saharan Africa. Women living with HIV face a higher incidence of HPV infections and severe health outcomes, increasing their cervical cancer risk [59]. Despite the need for early detection, screening programs in LMICs remain limited, unstructured, or completely absent due to resource constraints [60]. Many women lack basic information about cervical cancer prevention, vaccination, and screening, further contributing to late-stage diagnoses.

### Current Screening Methods for Cervical Cancer

The Visual Inspection with Acetic Acid (VIA) method is commonly used in LMICs, where nurses and midwives perform screenings [61]. However, VIA is highly subjective, with diagnostic accuracy dependent on the skill and experience of the healthcare provider. Patients with positive lesions identified through VIA are typically recommended for cryotherapy, but colposcopic-guided biopsies and pathology services are often unavailable [62]. Alternative screening methods such as Pap smears (cervical cytology) and HPV DNA testing provide greater accuracy, but high costs, a lack of trained pathologists, and limited government pathology labs restrict their use in LMICs [63].

### Gaps That Need to Be Filled

Limited access to screening facilities poses a significant challenge, as geographic barriers and high patient-to-provider ratios reduce availability, particularly for rural populations. Additionally, reliance on subjective screening methods such as VIA are widely used, but prone to inaccuracies due to its dependence on healthcare worker expertise. Furthermore, delays in diagnosis and treatment persist, as more effective methods like HPV DNA testing and Pap smears remain inaccessible to most patients due to cost and infrastructure limitations [62, 63].

### How AI is Filling Those Gaps

AI-driven cervical cancer screening can enhance diagnostic accuracy, streamline workflow efficiency, and improve early detection rates in LMICs. AI-augmented VIA screening can analyze colposcopy images to improve VIA accuracy and reduce reliance on human interpretation [64]. AI-based smartphone applications can facilitate automated lesion detection, making screening more accessible to remote areas [60]. Additionally, AI-driven risk stratification leverages HPV data to identify high-risk individuals, improving early detection and patient triage [64, 65]. Automated image analysis for cytology helps segment and classify images, reducing reliance on scarce pathologists while maintaining diagnostic accuracy. Moreover, AI-enhanced patient education and management, through chatbots and automated reminders, can increase screening uptake and ensure timely follow-ups.

### Ethical Issues of AI in Cervical Cancer Screening

Equitable access to AI-driven tools is essential, as AI must not exacerbate existing variations but instead ensure screening accessibility for all socioeconomic groups. Optimization for resource-constrained settings is also critical, requiring AI models to be lightweight and mobile-friendly to enable widespread adoption in low-resource environments [65]. Additionally, data privacy and security concerns must be prioritized, even in regions with limited AI governance frameworks, to protect patient information. Algorithmic validity across populations is another key factory, necessitating training on diverse, representative datasets to avoid predictive inaccuracies [66]. Finally, ensuring trust and explainability is vital, as AI models must be interpretable for clinicians to facilitate seamless integration into clinical workflows [67, 68].

### Strategies to Address Ethical and Implementation Challenges

Developing inclusive AI models requires training tools on globally diverse datasets to ensure accuracy across different demographic and genetic backgrounds. Implementing data protection standards is also important, as adherence to ethical AI frameworks and data privacy laws will ensure patient security in AI-driven screening [66]. Additionally, promoting clinician and patient trust is essential. AI models must be explainable and transparent, enabling healthcare providers to make informed decisions [68].

## AI and ML for Relapse Prevention in Schizophrenia

### Background

Schizophrenia is a severe mental illness affecting approximately 1% of the global population. As highlighted in the *Global Burden of Disease* study, schizophrenia ranks among the most debilitating health conditions [69]. Individuals living with schizophrenia face significantly reduced life expectancy, higher rates of poverty and homelessness, and profound social stigma and isolation [70]. These challenges are further exacerbated in lower-resource settings [71].

Despite these obstacles, early intervention, psychosocial support, and access to high-quality integrated mental health care can improve outcomes, enabling individuals with schizophrenia to manage symptoms and engage in meaningful activities. However, most people with schizophrenia lack access to the care they need, representing a significant implementation challenge. Key barriers include limited mental health workforce capacity with proper training and supervision, fragmented and poor-quality services that fail to meet patient needs, and lack of prioritization within health systems [71]. Emerging digital technologies, AI, and ML offer promising solutions to bridge these gaps in schizophrenia care [72, 73].

### AI and Digital Phenotyping for Relapse Detection

A notable case study demonstrating AI’s potential in schizophrenia care involved a smartphone-based digital phenotyping approach implemented in the United States and India [74]. In this study, AI and ML algorithms analyzed multiple data streams, including active data from ecological momentary assessments (EMAs) via smartphone surveys, and passive data such as activity levels, sleep patterns, and geolocation tracking. AI successfully detected early symptom changes and predicted relapse risk among schizophrenia patients. Participants were followed for up to 12 months, demonstrating feasibility across different global settings [75]. This AI-driven smartphone application has the potential to augment mental health services and enable real-time monitoring of patients. Digital phenotyping may help identify biomarkers predictive of treatment response and improved patient well-being.

### Challenges in AI Implementation for Schizophrenia Care

Despite AI’s promise, several challenges must be addressed before widespread adoption in routine schizophrenia care. One significant issue is with regards to technology access and connectivity. While smartphone ownership is increasing globally, continuous wireless data access can be prohibitive, and connectivity limitations in under-resourced settings may result in missing or low-quality data [76]. Additionally, there are concerns around algorithm generalizability and bias. The AI models used in the study were developed using U.S. patient data, and while these models were successfully applied in India, further refinement is needed with data from other global contexts [77]. In low-resource settings, phone sharing among family members may lead to misinterpretation of passively collected data. Ethical and privacy concerns further complicate the implementation of AI in schizophrenia care. AI’s ability to continuously collect sensitive patient data raises privacy and security issues, and ethical questions remain about whether continuous AI monitoring is acceptable in routine clinical care, particularly outside of research contexts [78]. Without proper safeguards, AI could exacerbate healthcare inequities by disproportionately benefiting well-resourced populations.

## AI in Tuberculosis Diagnosis

### Background

Tuberculosis (TB) remains a significant global health challenge, with 8.2 million new cases reported in 2023 [79]. The disease disproportionately affects LMICs, where men are 2.5 times more likely to be diagnosed than women [80]. Comorbidities such as HIV and diabetes further complicate disease management, increasing mortality risk and treatment failure rates [81, 82]. Despite advancements, traditional TB diagnostics, such as sputum smear microscopy, remain unreliable, particularly in resource-constrained settings, necessitating the development of innovative diagnostic technologies.

### Current TB Diagnostic Methods

Sputum smear microscopy is widely used in LMICs, but its low sensitivity and reliance on high-quality sputum samples limit its effectiveness [83]. Chest X-rays serve as an alternative, yet their interpretation depends on trained radiologists, who may be scarce in remote areas. Existing diagnostic approaches are often costly, time-intensive, and inaccessible for many TB-endemic regions. To bridge these gaps, there is an urgent need for scalable, affordable, and highly accurate TB screening solutions aligned with WHO’s target product profiles (TPPs) [84].

### Gaps That Need to Be Filled

Traditional TB screening methods are prone to inadequate diagnostics sensitivity and specificity, which can result in misclassification and lead to delayed or missed diagnoses [85]. Additionally, high risk populations are often excluded. Many patients, such as children and individuals with HIV, struggle to provide sputum samples, reducing diagnostic accuracy [81, 82]. Healthcare infrastructure barriers also play as significant role, as limited healthcare accessibility, weak public health infrastructure, and poverty contribute to delayed care and poor TB treatment outcomes [86].

### How AI is Filling Those Gaps

Computer-Aided Detection for Tuberculosis (CAD4 TB) is an AI-powered diagnostic tool that enhances TB screening efficiency and accessibility [87]. It automates chest X-ray interpretation by analyzing low-power, portable X-ray images and providing real-time, standardized TB screening, even in clinics without radiologists [88]. AI-driven pattern recognition also enhances screening accuracy and improves diagnostic precision, outperforming manual interpretations in resource-limited settings [89]. CAD4 TB’s compact design is scalable and portable, allowing for use in mobile health units and increasing reach in underserved communities [90]. By detecting pulmonary TB through imaging, CAD4 TB eliminates the need for sputum testing, making it more inclusive for populations that struggle to provide sputum samples. Additionally, the tool’s algorithm continuously improves and adapts over time, refining diagnostic accuracy across diverse populations and geographic regions.

### Ethical Issues of AI in TB Diagnosis

AI-based TB screening, such as CAD4 TB, presents several challenges related to data privacy, algorithmic reliability, and ethical considerations. The reliance on sensitive patient health data necessitates robust cybersecurity measures. Additionally, potential algorithmic distortions if the AI is trained on non-representative datasets, leading to inaccurate risk assessments and reinforcing diagnostic variation [91]. Ensuring transparency in AI decision-making is crucial, as AI-driven diagnostics must be explainable and interpretable to ensure clinical trust and accountability. Challenges in AI governance may arise because LMICs often lack regulatory frameworks for AI deployment, leading to potential mismanagement of health data [92]. Ethical concerns remain critical, and integration of AI must not replace human oversight to ensure patient-centered decision-making remains a priority.

## Strategies to Address Ethical and Implementation Challenges

Developing AI data governance frameworks, including cross-border data protocols and access controls ensures secure and ethical AI deployment. Enhancing algorithm diversity through regular dataset validation and retraining reduces predictive distortions and improves diagnostics. Additionally, ensuring explainability in AI systems by integrating transparent AI methodologies enhances clinical accountability, while homomorphic encryption safeguards patient data privacy.

As depicted in Table 1, we assessed each of the previous six case studies using the AI-Y Checklist. Case 1 used data from the Swedish National Study of Aging and Care (SNAC). When assessed using the AI-Y Checklist, Case 1 met the specified criteria and was marked as S (satisfactory) in topics 5 (contextual adaptability), 7 (privacy and data security), and 8 (transparency). It was not clear if topics 1 (model adaptivity), 4 (commercial interest assessment), or 6 (accessibility), were considered, so they were marked as U (unknown). The remaining topics 2 (accountability), 3 (AI development teams), 9 (targeted solutions), and 10 (generalizability) were not considered as part of the publication, and therefore marked as NS (not satisfactory).

Case 2 explores how in Europe, AI and ML are being used to improve environmental forecasting by enhancing the accuracy of weather predictions, early warning systems, and climate monitoring. These models address gaps in traditional methods by integrating diverse data sources and enabling real-time adaptability, which is especially important for public health preparedness. Efforts are also underway to improve model fairness, transparency, and inclusivity, particularly by incorporating data from underrepresented regions and promoting open science and stakeholder trust. To date, there are several ethical gaps in the development and deployment of AI-powered weather and climate models. Using the AI-Y Checklist, these models did not meet the criteria outlined in topics 3, 6, 8, or 9, and were marked as NS for these topics. Topics 1, 2, 4, 5, 7, and 10 were either not clearly addressed or not applicable, and thus marked as U.

In Case 3, Native-RISE project demonstrates how AI can support suicide risk identification in AI/AN communities in the U.S. by using locally sourced data and community-guided development. Led by JHCIH in partnership with Tribal leaders, the project trained a predictive model on 10 years of community data, achieving high accuracy and improving outreach to high-risk individuals. The model was co-developed with local providers and overseen by a community advisory board, ensuring cultural relevance, transparency, and ethical implementation. Native-RISE yielded strong results when assessed using the AI-Y Checklist. It satisfied the criteria for nearly all topics including 1, 2, 3, 4, 5, 7, 8, 9, and 10. Topic 6 was unknown, as the model has not yet been generalized to similar settings domestically or globally.

Furthermore, Case 4 shares how AI is being used in LMICs to improve cervical cancer screening by enhancing the accuracy of VIA and automating image analysis through mobile-friendly tools. These innovations help address challenges related to limited access, healthcare workforce shortages, and diagnostic subjectivity. AI applications also support patient education, risk stratification, and follow-up care, making screening more accessible and effective, particularly for women living with HIV in resource-constrained settings. In Uganda, an AI screening model was developed, trained, and validated based on mobile colposcopy images from different and diverse groups. It demonstrated contextual adaptability, equitable access, cultural responsiveness, data transparency, and privacy protection. The model satisfies the criteria in all the AI-Y Checklist topics, with the exception of topic 1 in which the model adaptivity is unknown.

In Case 5, a smartphone-based digital phenotyping tool was used to monitor individuals with schizophrenia and predict relapse risk using both active (e.g., in-app surveys) and passive (e.g., sleep, geolocation, activity) data. The AI model, developed in the U.S. and tested in both the U.S. and India, demonstrated feasibility across settings and supported early intervention. This approach offers a scalable solution to bridge mental health care gaps, particularly in low-resource environments. However, challenges around connectivity, data privacy, and cultural adaptation remain. Using the AI-Y Checklist, the AI digital phenotyping tool met the ethical criteria in topics 3, 4, 5, 6, 7, 8, 9, and 10, which were therefore marked as S. It was unclear if the criteria for topic 1, model adaptivity, were addressed, and topic 2, accountability, was not satisfied.

Case 6 describes CAD4 TB, an AI-powered diagnostic tool that automates the interpretation of chest X-rays to screen for TB, enabling rapid, accurate, and accessible diagnosis in resource-limited settings. It eliminates the need for sputum samples, especially beneficial for patients who cannot provide them, such as children and individuals with HIV, and can be deployed via portable, low-power devices in clinics lacking radiologists. The tool’s algorithm continuously improves with use, increasing diagnostic precision across diverse populations. While promising, ethical challenges around data privacy, algorithm bias, and regulatory oversight remain critical to address. Applying the AI-Y Checklist, AI for TB diagnosis satisfied the criteria outlined in topics 2, 6, 8, and 9. It was unclear if it met the criteria in topics 1, 4, 7, and 10, largely due to the vast and evolving nature of the work, and the topics were therefore marked as unknown. The criterion in topics 3 and 5 were not met and marked as NS. Topic 5 (contextual adaptability), for example, was limited due to challenges such as fuel restrictions preventing generators from powering the X-ray machine in the CAD4 TB study.

## Discussion

AI is playing an increasingly significant role in societies worldwide [93, 94], with applications spanning healthcare, finance, education, and other industries [95]. In healthcare, AI has the potential to enhance disease surveillance, improve diagnostic accuracy, optimize resource allocation, and strengthen health systems in LMICs [96–98]. These advancements align with efforts to achieve the Sustainable Development Goals and expand universal health coverage [99]. Given the rapid integration of AI in global health, previous studies have emphasized the need for clear standards and guidelines to ensure the responsible development and deployment of AI interventions [99, 100]. The AI-Y Checklist can address this need by providing AI developers, practitioners, and researchers with an actionable framework to evaluate the scientific rigor, transparency, and applicability of AI tools in diverse public health settings.

The case studies in this paper illustrate six examples of AI tools being applied to address public health challenges globally (seen in Table 1.). Each tool was assessed against the AI-Y Checklist, demonstrating how this standardized framework can support the evaluation and refinement of AI-based health technologies. A central theme across all case studies was the potential of AI to enhance healthcare delivery, particularly when models are developed using locally relevant data and are aligned with real-world health system needs. For example, Haroz highlighted how AI-supported clinical decision-making can improve the accuracy of suicide risk identification, particularly when models incorporate population-specific risk factors. Similarly, Nakatumba-Nabende discussed how AI-driven cervical cancer screening programs in LMICs could enhance diagnostic accuracy and accessibility, provided that implementation strategies account for infrastructural and operational constraints. Naslund further explored how AI-based tools in lower-resource settings can help address gaps in schizophrenia care, emphasizing the need to evaluate feasibility and community acceptability before widespread deployment. These examples build on existing literature demonstrating AI’s transformative potential in global health [99, 101, 102], while also underscoring implementation challenges that influence adoption and effectiveness in different populations.

Another critical issue identified across the case studies was the need for responsible AI deployment to ensure user protection. Fang examined AI applications in dementia care, emphasizing that privacy, validity, and transparency are essential for building trust and ensuring the ethical use of sensitive health data. Van Heerden similarly highlighted the importance of oversight in AI-driven TB management, particularly in LMICs where limited resources may hinder the enforcement of strong security and data governance protocols. To address this, AI systems designed for these regions must prioritize responsible data generation, the establishment of robust data infrastructures, and long-term strategies for data stewardship.

## Limitations of AI Based on the AI-Y Checklist

The application of AI-Y Checklist to the reviewed case studies highlights several limitations in the design and deployment of AI models. Representation remains a concern, while some models were retrained on localized datasets, many failed to explicitly address biases in data collection and model development. Historical accountability was largely absent, with most AI applications neglecting to account for or correct structural disparities in health data. In research and development, while a few projects engaged interdisciplinary teams, many lacked representation from affected community groups, limiting their contextual relevance. Commercial interests also influenced some AI models, with economic incentives potentially shaping their application rather than prioritizing equitable healthcare outcomes. Transparency issues were evident, as limited documentation and opaque decision-making processes reduced trust and accountability in AI-driven healthcare. Additionally, data privacy and security risks were insufficiently addressed, with many studies failing to implement or discuss strong privacy safeguards. Equitable access remains a challenge, as AI solutions developed in high-income settings often fail to consider accessibility barriers in low-resource environments. Finally, accountability in AI deployment was overlooked in many cases, with few studies implementing ongoing evaluation mechanisms to ensure AI remains aligned with ethical principles over time. These limitations underscore the need for rigorous AI evaluation frameworks to promote responsible and effective AI integration in healthcare.

## Limitations of AI Ethics in Public Health Applications

Despite growing recognition of ethical concerns in AI-driven healthcare, significant gaps persist. The evaluation of AI models using AI-Y Checklist reveals that many AI studies fail to address key ethical considerations such as model transparency, and accessibility. While AI holds promise for improving healthcare delivery, its ethical shortcomings raise concerns about unintended consequences, particularly across widespread populations.

## Limitations of This Review

This study employed a narrative review approach to map AI use cases against a structured ethical framework, providing valuable insights into emerging trends and highlighting research gaps. However, narrative reviews have inherent limitations that must be acknowledged. One major constraint is the lack of depth, as unlike systematic reviews that provide conclusive evidence, narrative reviews offer broad overviews that may lead to overgeneralizations. Additionally, potential selection bias is a concern, as the AI models analyzed were selected based on available literature, which may not fully capture the entire spectrum of AI-driven healthcare applications. The AI-Y Checklist also functions as a guideline rather than a definitive standard, meaning they are non-exhaustive, and additional ethical considerations may be relevant depending on the specific AI application and context. Furthermore, there is no fixed ethical threshold within the framework, as it does not establish a strict number of criteria that must be met for an AI tool to be deemed ethical. Instead, it serves as a flexible tool to promote ethical decision-making and highlight areas for improvement. These limitations emphasize the need for continued refinement of ethical evaluation frameworks to ensure comprehensive assessments of AI applications in healthcare.

## Prior Checklists

Established checklists from the literature have played a crucial role in standardizing research practices, enhancing transparency, and ensuring methodological rigor across various disciplines. In clinical research, the CONSORT (Consolidated Standards of Reporting Trials) statement has set the benchmark for reporting randomized controlled trials, improving the clarity and reliability of trial findings [103, 104]. Similarly, the STROBE (Strengthening the Reporting of Observational Studies in Epidemiology) guidelines have provided a structured approach for reporting observational studies, ensuring comprehensive documentation of study design, data sources, and limitations [105, 106]). In environmental sciences, the FAIR (Findable, Accessible, Interoperable, and Reusable) principles have established best practices for data management, facilitating data-sharing and reproducibility in AI-driven environmental modeling [107]. Additionally, in AI ethics, the ACM Principles for Algorithmic Transparency and Accountability have emphasized the need for explainability, bias mitigation, and human oversight in algorithmic decision-making [108]. These checklists provide a structured foundation for evaluating research quality and integrity, underscoring the need for similar structured frameworks in AI-driven health applications. As AI research continues to advance, the need for structured assessment tools becomes increasingly apparent. The checklist not only facilitates a consistent evaluation of AI-driven technologies but also ensures that these innovations align with ethical and scientific principles. The AI-Y Checklist builds upon established evaluation frameworks by introducing a structured approach specifically designed for assessing AI applications in public health. Unlike existing checklists focused on clinical trials or data management, this framework integrates key AI-specific considerations—such as model adaptivity, ethical accountability, and contextual adaptability, thereby ensuring that AI-driven technologies align with real-world health system needs and ethical standards.

## Conclusion

Despite AI’s potential to enhance healthcare systems, its ethical shortcomings remain a major challenge. The application of the AI-Y Checklist reveals persistent gaps in bias mitigation, accountability, transparency, and equitable deployment. Moving forward, AI developers and public health practitioners must prioritize ethical design principles and implement standardized frameworks to ensure AI technologies benefit all populations equitably.

The AI-Y Checklist offer a structured approach for guiding the development, implementation, and evaluation of AI-based digital health technologies. This framework is designed to be broadly applicable, practical, and actionable, ensuring that AI tools are rigorously tested and appropriately contextualized before integration into public health systems. Establishing clear guidelines for AI in healthcare is essential to achieving digital inclusion, which is a fundamental component of digital literacy. Digital literacy has been referred to as a"super social determinant of health"due to its influence on multiple factors affecting population health [109, 110]. By adopting standardized evaluation criteria, the public health and AI communities can work toward ensuring that AI-driven innovations effectively contribute to improved health outcomes on a global scale.

## Key References

- Haroz EE, Rebman P, Goklish N, et al. Performance of Machine Learning Suicide Risk Models in an American Indian Population. *JAMA Netw Open*. Oct 1 2024;7(10):e2439269.

- ⚬ Machine learning models outperformed existing suicide screening methods in predicting suicide risk among American Indian patients, highlighting the importance of culturally validated tools.

- Cohen A, Naslund J, Lane E, et al. Digital phenotyping data and anomaly detection methods to assess changes in mood and anxiety symptoms across a transdiagnostic clinical sample. *Acta Psychiatr Scand*. Mar 2025;151(3):388–400. doi: 10.1111/acps.13712

- ⚬ Smartphone-based digital phenotyping using active and passive data can predict mood and anxiety symptom changes across diverse clinical populations.

- Wei D, Freydenzon A, Guinebretiere O, et al. Ten years preceding a diagnosis of neurodegenerative disease in Europe and Australia: medication use, health conditions, and biomarkers associated with Alzheimer's disease, Parkinson's disease, and amyotrophic lateral sclerosis. *EBioMedicine*. 2025;113.

- ⚬ AI-powered anomaly detection of medications, health conditions, and biomarkers predicted the risk of Alzheimer's, Parkinson's, and ALS up to ten years before diagnosis.

## Data Availability

No datasets were generated or analysed during the current study.
